# MitoCeption as a new tool to assess the effects of mesenchymal stem/stromal cell mitochondria on cancer cell metabolism and function

**DOI:** 10.1038/srep09073

**Published:** 2015-03-13

**Authors:** Andrés Caicedo, Vanessa Fritz, Jean-Marc Brondello, Mickaël Ayala, Indira Dennemont, Naoill Abdellaoui, Florence de Fraipont, Anaïck Moisan, Claire Angebault Prouteau, Hassan Boukhaddaoui, Christian Jorgensen, Marie-Luce Vignais

**Affiliations:** 1 IRMB CHU Saint Eloi, 80 rue Augustin Fliche, 34295 Montpellier cedex 5, University of Montpellier, France; 2 Inserm U1183,CNRS UMR 5535/IFR122, 1919 route de Mende, 34293 Montpellier Cedex 5, University of Montpellier, France; 3 Institut de Génétique Moléculaire de Montpellier, CNRS UMR 5535/IFR122, 1919 route de Mende, 34293 Montpellier Cedex 5, University of Montpellier, France; 4 Univ Grenoble Alpes, 38000 Grenoble, France; 5 Inserm, U823 and U836, 38000 Grenoble, France; 6 Grenoble University hospital, Institut of Biologie and Pathology, 38000 Grenoble, France; 7 French blood company/Grenoble University hospital, Cell Therapy Unit, 38000 Grenoble, France; 8 Inserm U1051, Montpellier, France; 9 Department for Biotherapy at CHU Lapeyronie University Hospital, Montpellier, France

## Abstract

Mitochondrial activity is central to tissue homeostasis. Mitochondria dysfunction constitutes a hallmark of many genetic diseases and plays a key role in tumor progression. The essential role of mitochondria, added to their recently documented capacity to transfer from cell to cell, obviously contributes to their current interest. However, determining the proper role of mitochondria in defined biological contexts was hampered by the lack of suitable experimental tools. We designed a protocol (MitoCeption) to directly and quantitatively transfer mitochondria, isolated from cell type A, to recipient cell type B. We validated and quantified the effective mitochondria transfer by imaging, fluorescence-activated cell sorting (FACS) and mitochondrial DNA analysis. We show that the transfer of minute amounts of mesenchymal stem/stromal cell (MSC) mitochondria to cancer cells, a process otherwise occurring naturally in coculture, results in cancer cell enhanced oxidative phosphorylation (OXPHOS) activity and favors cancer cell proliferation and invasion. The MitoCeption technique, which can be applied to different cell systems, will therefore be a method of choice to analyze the metabolic modifications induced by exogenous mitochondria in host cells.

Mitochondria are involved in the central cell tasks of nutrient uptake and energy production. They are therefore at the core of a number of essential biological functions and corresponding disorders^1^,^2^,^3^,^4^. Mitochondria are also actively involved in cancer progression, including metastasis, and in acquired resistance to therapy^5^,^6^,^7^,^8^. These biological functions associated with a better understanding of the mitochondria dynamics and signaling have triggered a renewed interest in the field^2^,^4^,^9^. Interestingly, in the past few years, several laboratories have reported the capacity of mitochondria to be transferred between cells, through nanotube formation, leading to cellular reprogramming and to phenotypes as diverse as protection against tissue injury and resistance to therapeutic agents^10^,^11^,^12^,^13^,^14^,^15^,^16^,^17^. These first *in vitro* observations of the mitochondria transfer were confirmed *in vivo* and factors involved in the trafficking of mitochondria through nanotubes, notably the connexin 43 and the mitochondrial Ca^2+^-binding GTPase Miro1 (RHOT1), were identified^10^,^18^. A number of these mitochondria transfers were shown to originate, through the formation of nanotube structures, from mesenchymal stem/stromal cells (MSCs) and to target various tissues, leading to the transfer of MSC mitochondria to cardiomyocytes, endothelial cells, pulmonary alveolar epithelial cells, renal tubular cells and cancer cells^10^,^11^,^12^,^14^,^19^,^20^,^21^. These various studies clearly showed that MSC mitochondria could convey new properties to the recipient cells.

MSCs are identified by a panel of receptors, notably CD71+, CD90+, CD105+, CD45-, CD34-, and characterized by their immunosuppressive properties and their capacity to differentiate to different lineages^22^,^23^. MSCs are recruited to inflammatory sites where they can contribute to tissue repair. They are also recruited to tumor sites where they can modify cancer cell growth and metastatic potential as well as response to therapy^24^,^25^,^26^,^27^,^28^,^29^,^30^,^31^,^32^,^33^,^34^,^35^,^36^,^37^. In addition to the long-known cytokine-dependent communications between the stromal and cancer cells^38^, current data indicate that metabolite exchange and direct cell-cell contacts also greatly contribute to these effects, through cancer cell metabolic reprogramming^5^,^11^,^39^,^40^. As previously shown by others^11^, and as we show in this manuscript, MSCs can transfer mitochondria to cancer cells. Since MSCs are part of the cancer cell microenvironment, this can open new routes for cancer cell metabolic reprogramming with functional consequences for tumor progression and resistance to anti-cancer drugs.

Despite the obvious interest of this novel means of cell-cell communication, the precise characterization of MSC mitochondria effects on the recipient cells remained partly elusive because of the lack of suitable study systems. Technical approaches to artificially transfer mitochondria from donor to recipient cells have been sought in the past. This was achieved by direct injection of mitochondria into oocytes^41^,^42^,^43^. The specific contribution of mitochondrial DNA (mtDNA) was also studied by preparing transmitochondrial cybrids. These cybrids are the result of the fusion of enucleated cells, whose mtDNA is to be analyzed, with ρ° cells that are deficient in mtDNA^44^,^45^. However, these techniques are complex and difficult to put into practice for large cell populations.

We developed a model system whose goal is to study the interactions between human mesenchymal stem cells (hMSCs) and MDA-MB-231 cancer cells. In addition to the cytokine-mediated communication and the metabolite exchange^5^,^38^,^39^,^40^,^46^, we show herein that MSCs can transfer mitochondria to the MDA-MB-231 cancer cells. To distinguish the effects of MSC mitochondria from other signaling contributions, we designed a method (MitoCeption) for quantitatively transferring MSC mitochondria to the MDA-MB-231 cells, in amounts comparable to those occurring in coculture. We exploited differences found in the mitochondrial DNA (mtDNA) sequences between donors of MSCs and donors of cancer cells to specifically quantify the mtDNA of each cell origin. We demonstrated that the MSC mitochondria transferred by MitoCeption have the capacity to increase the MDA-MB-231 endogenous pool of mitochondria and that acquisition of these MSC mitochondria by the MDA-MB-231 cells result in an enhancement of both the energetic metabolism and the functional properties of the targeted MDA-MB-231 cells.

## Results

### MSCs transfer mitochondria to cancer cells

**Figure 1:**
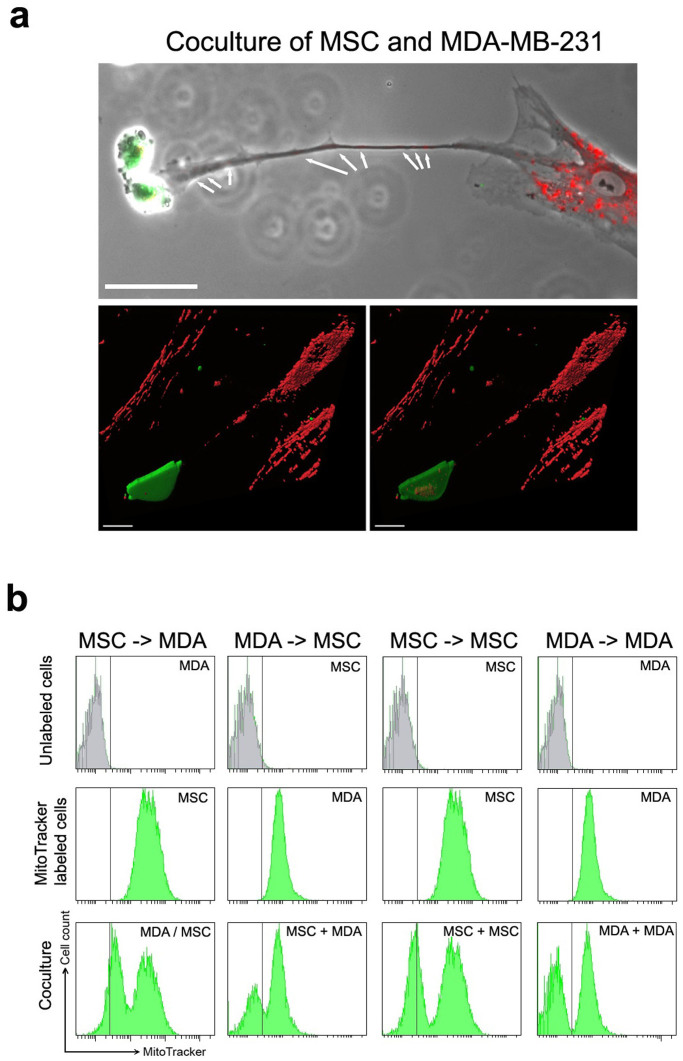
Exchange of mitochondria between hMSCs and MDA-MB-231 cancer cells. (a) Coculture (24 h) of human MSCs (hMSCs) (red MitoTracker prestained) and MDA-MB-231 cells (green CellTracker prestained). Upper panel, fluorescence and phase contrast, MSC mitochondria indicated by arrows (scale bar, 50 μm). Lower panels, 3D reconstructions of confocal images (scale bar, 20 μm). (b) FACS analysis of the transfer of mitochondria between hMSCs and MDA-MB-231 cancer cells. MSCs were MitoTracker-labeled 24 hours beforehand and cocultured with the unlabeled MDA-MB-231 cells for 24 hours to check their capacity to transfer mitochondria to the cancer cells (left column, MSC to MDA). Likewise, the three other cell combinations (transfer of mitochondria from MDA to MSC, MSC to MSC and MDA to MDA) were tested.

Time-lapse imaging of cocultures of CellTracker labeled MSCs and MDA-MB-231 cancer cells had shown that MSCs could make strong physical interactions and transfer cellular components to MDA-MB-231 cancer cells (Supplementary Fig. 1). To check whether mitochondria were involved in this transfer, MSCs were stained with a MitoTracker and incubated with unlabeled MDA-MB-231 cells for 24 hours before imaging. We showed that MSCs could form long mitochondria-containing protrusions targeting the MDA-MB-231 cancer cells and leading to the transfer of MSC mitochondria (Fig. 1). Furthermore, the transfer of MSC mitochondria to cancer cells was also demonstrated in heterologous cocultures between human MSCs and murine TSA-pc cancer cells, using an antibody (Ab-2) specific for human mitochondria (Supplementary Fig. 2). We controlled that the MitoTracker signal observed in the cancer cells when in coculture with the MitoTracker-labeled MSCs was not due to the leakage of the MitoTracker dye from the MSCs. For that, we checked that incubating MDA-MB-231 cells with a conditioned medium from the MitoTracker-labeled MSCs did not lead to a MitoTracker signal in the cancer cells (data not shown). Importantly to prevent such MitoTracker leakage, we found it necessary to avoid incubation of the MitoTracker-labeled MSCs with EDTA containing solutions. The transfer of mitochondria from hMSCs to the MDA-MB-231 cells (24 hour-coculture with a 1:1 cell ratio) was confirmed by Fluorescence Activated Cell Sorting (FACS), on the basis of the acquired MitoTracker staining by the MDA-MB-231 cancer cells after their incubation with the MitoTracker-prelabeled MSCs (Fig. 1b, left column). The other cell combinations showed that MSCs could transfer mitochondria among themselves and that MDA-MB-231 cells could transfer mitochondria to MSCs, with lower efficacy, and not to themselves (Fig. 1b). These results highlighted the complex interactions between MSCs and cancer cells. To distinguish the effects of the transferred MSC mitochondria from MSC secreted factors, we designed a protocol (MitoCeption) for the quantitative transfer to the cancer cells of mitochondria isolated from hMSCs.

### Validation of the MitoCeption methodology

**Figure 2:**
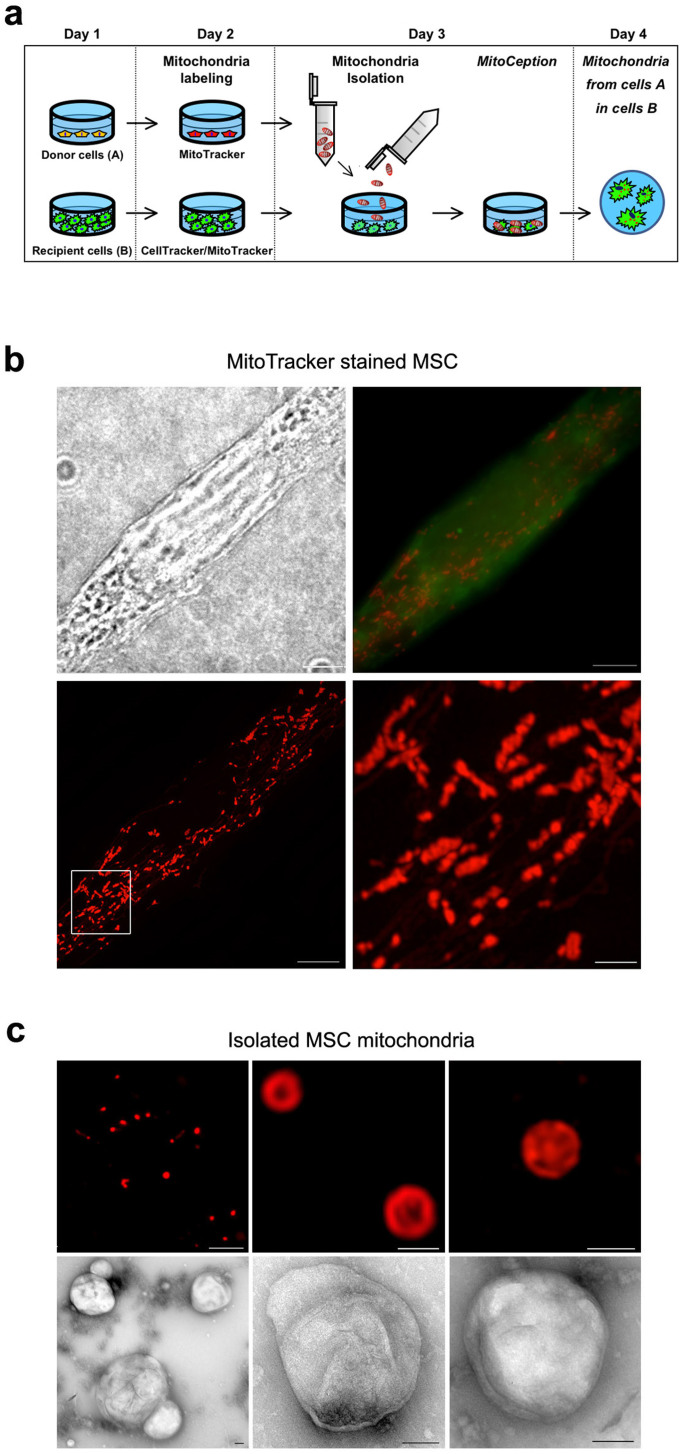
Transfer of mitochondria isolated from MSCs to target cells by MitoCeption. (a) Timeline for the generation of MitoCepted cells. (b) Imaging of MSC mitochondria. MSCs were prestained with a green CellTracker and a red MitoTracker, fixed and mounted in ProLong Gold before their super-resolution imaging with a OMX microscope. Top panels: transmission imaging and overlay of the CellTracker and MitoTracker signals (wide field). Lower panels: super-resolution imaging of the MSC mitochondria network with an enlarged view of the region of interest. Scale bars, 5 μm, 1 μm in the magnification panel. (c) Imaging of the mitochondria isolated from MSCs. Top panels: the mitochondria isolated from the red MitoTracker-labeled MSCs were directly mounted in PBS before their super-resolution imaging with the OMX microscope. Scale bars, 5 μm in left panel, 0.5 μm in the right panels. Lower panels: the MSC mitochondria preparation (unlabeled) was analyzed by transmission electron microscopy under negative staining conditions. Different fields are shown. Scale bars, 100 nm.

The MitoCeption protocol allows the transfer of mitochondria isolated from cell type A to cell type B so that, at the end, cell type B contains both its own and the exogenous mitochondria (Fig. 2a). To follow the mitochondria transfer process, MSCs were MitoTracker labeled 24 hours before their isolation and their transfer to the MDA-MB-231 cells, so that excess MitoTracker dye could be washed out before the mitochondria isolation and transfer. We used the super-resolution 3 dimensional-Structured Illumination Microscopy (3D-SIM), which improves resolution by a factor of two in x, y and z^47^, to analyze MSC mitochondria both in the MSCs and after their isolation from MSCs. For cell imaging, MSCs were fixed and mounted in ProLong Gold several days before the 3D-SIM imaging. The MSC mitochondria were found spread throughout the cell and enlargement of the field showed that mitochondria of different sizes and both as single and multiple units could be observed (Fig. 2b). After their isolation, the MitoTracker-labeled MSC mitochondria were similarly checked by super resolution microscopy (3D-SIM). In that case though, mitochondria were immediately imaged in DMEM (without red phenol), without prior fixation. The isolated mitochondria were found as round organelles, with detectable MitoTracker stained structures and with a diameter in the range of 0.5 μm (Fig. 2c, top panels). The mitochondria preparation (without prior MitoTracker staining) was also analyzed by transmission electron microscopy (TEM), with uranyl acetate negative staining. In that case too, imaging was performed immediately following the mitochondria isolation. Negative staining TEM allowed determining the shape and size of the isolated mitochondria, with minimal treatment of the mitochondria sample (Fig. 2c, lower panels). Mitochondria were found as organelles of different sizes and shapes, reminiscent of what was observed in the cell, with a mean diameter measured for the mitochondria by TEM of 476 ± 33 nm. Remarkably, the average sizes of the isolated mitochondria were found to be similar with the two imaging techniques, relying either on fluorescent labeling (3D-SIM) or on unlabeled mitochondria (TEM).

**Figure 3:**
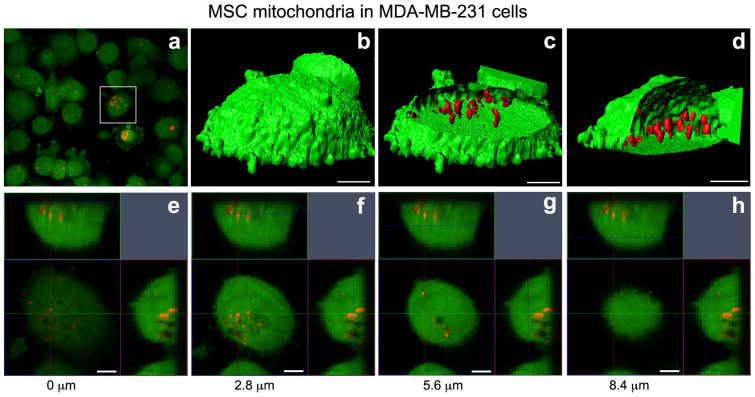
MDA-MB-231 cell imaging after the transfer by MitoCeption of mitochondria isolated from MSCs to MDA-MB-231 cells. Red MitoTracker-labeled MSC mitochondria were transferred to MDA-MB-231 cells prelabeled with a green CellTracker. (a) 2D view of the culture after the mitochondria transfer. (b–h) Stacks of confocal images were made on the cell shown in (a) with a multiphoton microscope. (b–d) A 3D reconstruction of the cell was made. Shown are the cell isosurface views (Imaris) (b), with either a xy plane section (c) or a yz plane section (d), scales, 5 μm. (e–h) Four individual confocal sections are shown, with the corresponding orthogonal views, starting at 0 μm (cell bottom) and spanning the cell height. The level of each confocal image is shown on the corresponding orthogonal views. Scale bars, 5 μm.

**Figure 4:**
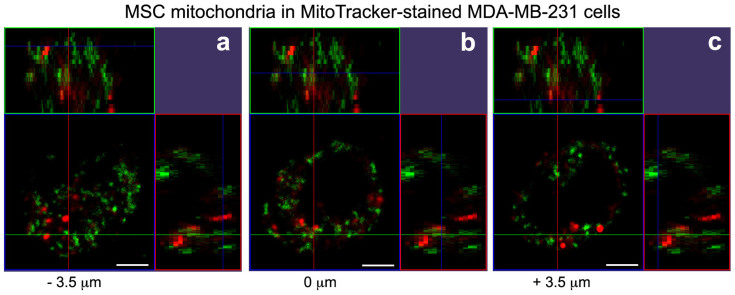
Mitochondria imaging in the MDA-MB-231 cells after the transfer by MitoCeption of mitochondria isolated from MSCs to the MDA-MB-231 cells. Red MitoTracker-labeled MSC mitochondria were transferred to MDA-MB-231 cells prelabeled with a green MitoTracker. Shown are confocal sections and the corresponding orthogonal views, made with a multiphoton microscope, in the middle of the endogenous MDA-MB-231 mitochondria network (b), 3.5 μm beneath (a) and 3.5 μm above (c). Scale bars, 5 μm.

For MitoCeption, the MSC mitochondria were transferred to the MDA-MB-231 cancer cells following the protocol described in the methods section. In particular, the culture plates were centrifuged immediately after the addition of the MSC mitochondria to curtail the time spent by the mitochondria in the warm culture medium, prior to their interaction with the target cells, with the goal of limiting mitochondria damage. To check the entry of the MSC mitochondria inside the target cancer cells, the cells were rinsed with the culture medium twenty-four hours after the mitochondria transfer. The live cells were immediately analyzed by confocal imaging, using a multiphoton microscope (Fig. 3 and Fig. 4). In figure 3, the MDA-MB-231 cells were stained with a green CellTracker prior to the MitoCeption with the red MitoTracker-labeled MSC mitochondria. Fluorescence imaging showed that most MDA-MB-231 cells contained signals corresponding to MSC mitochondria (Fig. 3a). To further determine the localization of the MitoTracker stained MSC mitochondria, we generated a stack of confocal images, 0.7 μm apart, focusing on a MDA-MB-231 cell with an apparently high MSC mitochondria content (Fig. 3a, framed cell). The 3D cell reconstruction with the accompanying orthogonal sections (Fig. 3b–d) showed that MSC mitochondria were found inside the cancer cell, as shown also by a selection of the confocal images used for this 3D reconstruction (Fig. 3e–h). To determine the position of the MitoCepted MSC mitochondria relative to the endogenous MDA-MB-231 mitochondria, the MDA-MB-231 cells were stained with a green MitoTtracker prior to the MitoCeption with the red MitoTracker-labeled MSC mitochondria. Confocal sections and the corresponding orthogonal views show that the transferred MSC mitochondria were found close to the endogenous MDA-MB-231 mitochondria network, and thus further confirmed their localization inside the MDA-MB-231 cancer cells (Fig. 4a–c).

In a first approach to characterize these transferred mitochondria, we tested whether they could, like the endogenous MSC mitochondria, transfer to the MDA-MB-231 cells in coculture, We showed that these (red MitoTracker stained) MitoCepted mitochondria spread out among the endogenous MSC mitochondria and that, following coculture with the MDA-MB-231 cells, they could transfer to the cancer cells as did the endogenous MSC mitochondria (Supplementary Fig. 3). Mitochondria transfer by the MitoCeption protocol was also achieved from MSCs to non-adherent cells, like Jurkat cells, and among cancer cells (data not shown).

### Quantification of the transfer of mitochondria by MitoCeption

**Figure 5:**
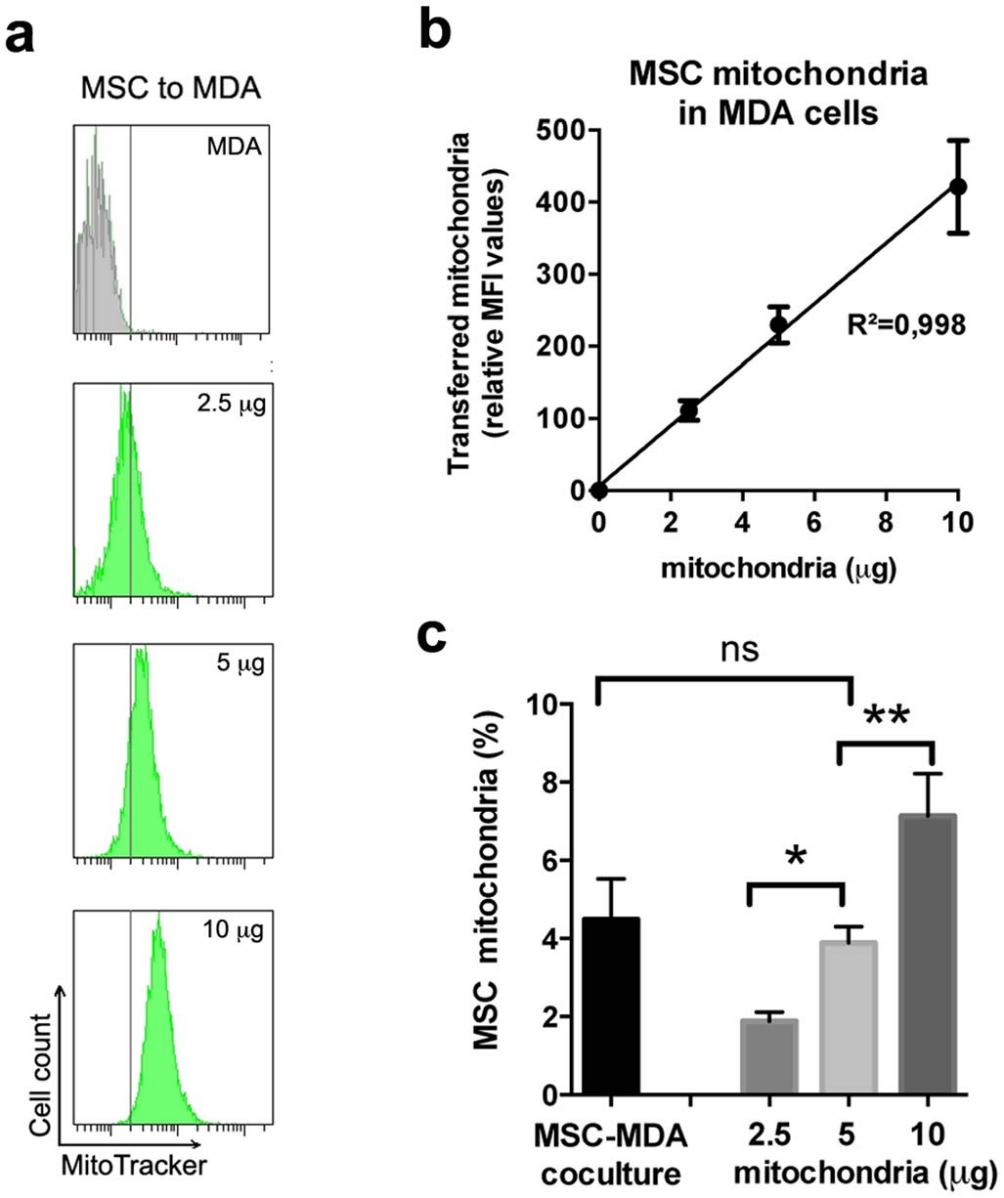
FACS analysis of MDA-MB-231 cells MitoCepted with increasing amounts of mitochondria (expressed as μg of proteins) isolated from MitoTracker-labeled MSCs. (a) Representative FACS experiment. (b) Relative MFI values (n = 8) (3 MSC donors). (c) Amounts of transferred MSC mitochondria relative to the endogenous MDA-MB-231 mitochondria, after coculture (ratio 1:1) or MitoCeption (n = 7). All values are shown as mean ± S.E.M. Linear regressions are shown.

FACS analysis was used to quantify the efficiency of the mitochondria transfer, as described above, and the MSC mitochondrial DNA (mtDNA) concentration was estimated by qPCR. FACS analysis showed a dose-dependent uptake of MSC mitochondria by the MDA-MB-231 cancer cells (Fig. 5a, b). Interestingly, the MSC mitochondrial mass detected in the cancer cells after the coculture (1:1 ratio between MSCs and MDA-MB-231 cells) was similar to that following MitoCeption (condition 5 μg), respectively 4.5% ± 1.0 and 3.9% ± 0.4 relative to the endogenous MDA-MB-231 mitochondria (Fig. 5c).

**Figure 6:**
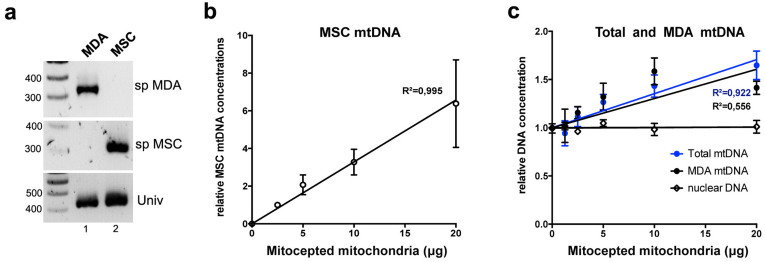
Mitochondrial DNA (mtDNA) PCR quantification following MitoCeption. (a) PCR amplification products of MDA-MB-231 and MSC mtDNA using primers specific for each cell type or universal (agarose gel, markers in bp). (b) MSC mtDNA quantification following MitoCeption to MDA-MB-231 cells (n = 6). (c) Quantification of total mtDNA, mtDNA of MDA-MB-231 origin and nuclear DNA in MDA-MB-231 cells after MitoCeption of MSC mitochondria (n = 6, 3 MSC donors). Values are expressed as fold relative to MDA-MB-231 cells without MSC mitochondria. All values are shown as mean ± S.E.M. Linear regressions are shown.

The concentrations of transferred MSC mitochondrial DNA (mtDNA) into the cancer cells were quantified by qPCR. Mutations present in the D-loop of the MDA-MB-231 mtDNA were used to distinguish mtDNA originating from MSCs or from the cancer cells and to design primers for the specific amplification of mtDNA from either cell type (Fig. 6a). Likewise, mtDNA from different MSC donors could also be distinguished by targeting specific SNPs (data not shown). As expected, MSC mtDNA concentrations increased in a dose dependent fashion, following MSC mitochondria transfer in the cancer cells (Fig. 6b). The total (endogenous plus exogenous) mtDNA concentration was also found to augment with 1.11 ± 0.10, 1.27 ± 0.08, 1.43 ± 0.11 and 1.65 ± 0.15 fold increases for the respective added amounts of mitochondria of 2.5, 5, 10 and 20 μg (Fig. 6c). Unexpectedly, however, this increase was not due to the MSC mtDNA, found in minute amounts compared to the endogenous MDA-MB-231 mtDNA, respectively of 0.21% ± 0.09, 0.43% ± 0.11, 0.69% ± 0.14 for the 2.5, 5 and 10 μg conditions. Instead, it was due to increased amounts of the endogenous mtDNA, as shown using PCR primers specific for the MDA-MB-231 mtDNA (Fig. 6c). This suggested that the acquired MSC mitochondria could induce an increase in the endogenous mtDNA concentrations, possibly through the enhancement of the endogenous mtDNA replication or by inhibition of its degradation.

### Effect of MSC mitochondria on cancer cell metabolism and functional properties

**Figure 7:**
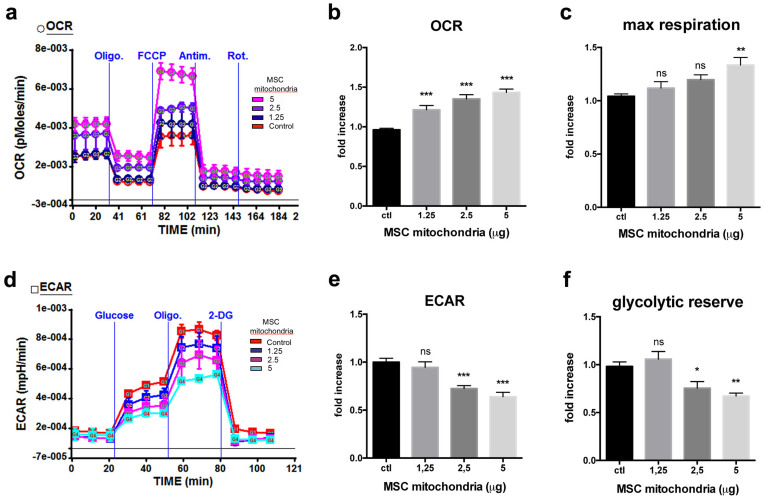
Effects of MSC mitochondria on MDA-MB-231 metabolism. All values are expressed as a fold of the control MDA-MB231 cells. The Extracellular Flux analysis of the MDA-MB-231 cells MitoCepted with increasing amounts of MSC mitochondria (μg) was performed. (a) OXPHOS activity. Measurements of oxygen consumption rates (OCR, pMoles O_2_/min) were performed in quadruplicates in 6 experiments (2 MSC donors). (b) Extracellular Acidification Rates (ECAR, mpH/min) were measured in quadruplicates in 3 experiments (2 MSC donors). All values are shown as mean ± S.E.M.

**Figure 8:**
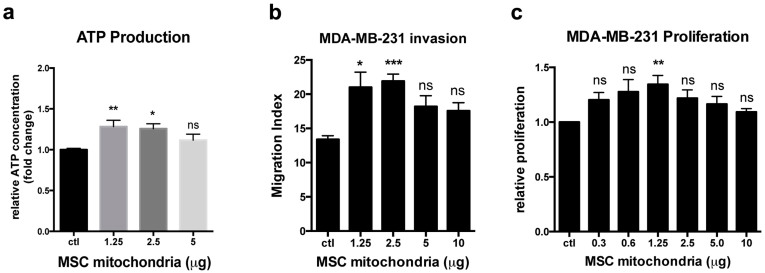
Effects of MSC mitochondria on MDA-MB-231 ATP production, invasion and proliferation capacities. (a) The level of total ATP in MDA-MB-231 cells MitoCepted with MSC mitochondria was measured by a chemoluminescent assay and the values of Relative Luciferase Units (RLU) obtained are expressed as fold of the control MDA-MB-231 cells (n = 8). (b) To measure the effect of the MitoCepted MSC mitochondria on the MDA-MB-231 invasion capacities, MDA-MB-231 cells were seeded in a 3D-collagen matrix and let to migrate for 72 hours. The invasion index represents the weighted migration, taking into account the number of cells that migrated at 25 and 50 μm (n = 20, 2 MSC donors). (c) For the proliferation assay, cell numbers were determined after 5 days of culture (n = 6, done in quadruplicates, 3 MSC donors). All values are shown as mean ± S.E.M.

We next investigated the effect of MSC mitochondria on MDA-MB-231 cancer cell metabolic activity. For all analyses of the functional effects of the MSC mitochondria inside the cancer cells, only unlabeled MSC mitochondria were used in order to avoid any possible bias due to the MitoTraker staining. After MitoCeption, the MDA-MB-231 cancer cells containing different amounts (in μg) of MSC mitochondria were trypsinized and transferred to the SeaHorse plates for Extracellular Flux analysis, at a concentration such that cells were confluent on the day of the SeaHorse measure. Oxidative phosphorylation (OXPHOS) and glycolysis activities were measured 48 hours after the transfer of MSC mitochondria and all activities were normalized to the number of cells present in the wells, as counted immediately after the SeaHorse measures. For OXPHOS, the oxygen consumption rate (OCR) was measured under basal conditions and in response to mitochondrial inhibitors. The optimal concentration for the FCCP uncoupler was determined for the control condition and for each of the mitochondria concentrations tested (1.25, 2.5 and 5 μg) (Supplementary Fig. 4). The FCCP concentration of 0.33 μM allowed the maximal oxygen consumption rate for all MitoCeption conditions and for the control MDA-MB-231 cells and was therefore used for all experiments. Upon MitoCeption of MSC mitochondria, the basal OCR values of the cancer cells were significantly increased, in a dose-dependent manner, with respectively 1.22 ± 0.05, 1.35 ± 0.05 and 1.43 ± 0.04 fold increases for the 1.25, 2.5 and 5 μg conditions (Fig. 7a,b). Moreover, the maximal respiration was also significantly enhanced for the 5 μg condition, with a 1.34 ± 0.07 fold increase (Fig. 7c). This indicated that MSC mitochondria acquisition could modify the metabolic activity of the cancer cells by increasing their respiratory capacity. In contrast, both the extracellular acidification rate (ECAR), indicative of the glycolytic activity, and the glycolytic reserve were decreased (respectively 0.64 ± 0.05 and 0.67 ± 0.03 fold of the control, for the 5 μg condition) (Fig. 7d–f). Production of ATP by the MDA-MB-231 cells after MitoCeption was also measured. Importantly, acquisition of MSC mitochondria by MDA-MB-231 cells resulted in significant increases in ATP production (1.28 ± 0.08 and 1.26 ± 0.06 fold increases for respectively the 1.25 and 2.5 μg conditions) (Fig. 8a). We also checked the effect of MSC mitochondria on MDA-MB-231 cells functional capacities, notably migration and proliferation. Using a 3D-collagen invasion assay, we showed that acquisition of MSC mitochondria by the cancer cells enhanced their invasion capacity 1.6 fold, with a migration index raising from 13.4 ± 0.5 for the control cells to 21.9 ± 1.0 for the 2.5 μg condition, within the 3 day migration time-frame (Fig. 8b). MDA-MB-231 cell proliferation was also stimulated, as measured over a 5-day period following MSC mitochondria acquisition, with direct cell counting as a readout (1.35 ± 0.08 fold increase for the 1.25 μg condition, Fig. 8c). Interestingly, although both the invasion and proliferation capacities of the MDA-MB-231 cells were increased upon acquisition of MSC mitochondria, these effects were not observed for higher amounts of transferred MSC mitochondria, suggesting deleterious effects of excess MSC mitochondria for the cancer cells.

## Discussion

The transfer of mitochondria from donor to recipient cells constitutes a new communication process, that has been observed for different cell systems by a number of research teams^10^,^11^,^12^,^13^,^14^,^15^,^16^,^17^,^18^,^19^,^20^,^21^. The biological consequences and the mechanisms of these nanotube-dependent mitochondria transfers are just beginning to emerge. We devised a protocol, MitoCeption, that allows the transfer of mitochondria, isolated from the MSC donor cells, to the MDA-MB-231 target cancer cells. We demonstrated a dose-dependent uptake of the MSC mitochondria by the MDA-MB-231 target cells following MitoCeption. By specific mtDNA tracking, we showed that the acquisition of the MSC mitochondria (isolated beforehand) by the MDA-MB-231 cells led to a notable increase in the MDA-MB-231 mtDNA concentration, by mechanisms that still need to be established. Overall, this resulted in increased OXPHOS, ATP production and both invasion and proliferation capacities for the MDA-MB-231 cancer cells.

We used high-resolution imaging to estimate the quality of our preparations of MSC mitochondria and to compare them with the endogenous mitochondria. Both the endogenous and isolated MSC mitochondria appeared heterogeneous in size and shape. The diameter range of the isolated mitochondria was of 0.5 μm as observed both by 3D-SIM super resolution imaging and negative staining transmission electron microscopy. Note worthily, mitochondria in the fixed cells appeared of smaller dimensions than the isolated mitochondria. Among several hypotheses, the PFA fixation and most of all the several day-dehydration following the Prolong Gold cell mounting, required for the 3D-SIM super resolution imaging of the cells, could be responsible for this size difference between the fixed and freshly isolated mitochondria. To check that the MSC mitochondria were internalized inside the MDA-MB-231 cells following MitoCeption, we used MSC mitochondria that had been MitoTracker stained before their isolation. The 3D imaging of the cells showed that the MitoCepted MSC mitochondria were found inside the MDA-MB-231 cells, and not at the cell membrane for instance, and that moreover they were spread out throughout the endogenous MDA-MB-231 mitochondria network.

We did not observe fusion between the transferred and endogenous mitochondria. Several hypotheses can be proposed for these observations. First, in order to avoid biases linked to a possible leak of the MitoTracker dyes, we worked in stringent conditions that may possibly prevent us from observing the mitochondria fusion, taking place after MitoCeption of the MSC mitochondria. Alternatively, the membrane potential of the isolated mitochondria might be modified through their isolation and transfer process, which would have an effect on the rate of fusion with the endogenous mitochondria. As a third hypothesis, it is known that high glucose can induce mitochondria fragmentation^48^. In our experiments, MSC were cultured in low glucose αMEM and then transferred to MDA-MB-231 cells cultured in high glucose DMEM. Even though the kinetics described so far for this glucose-dependent mitochondria fragmentation phenomenon are rapid, a change in the glucose concentration might also have an effect on the capacity of the transferred MSC mitochondria to fuse the MDA-MB-231 mitochondria. MitoCeption might provide a useful tool to study these processes.

We observed that the acquisition of the isolated MSC mitochondria, even in low amounts, by the MDA-MB-231 cells resulted in an increase in MDA-MB-231 mitochondrial DNA concentrations, OXPHOS activity and ATP production. Several mechanisms, non-exclusive one to the other, could account for these effects. First of all, it could be due to the OXPHOS activity of the transferred MSC mitochondria or to their signaling through the production of metabolites or adenine nucleotides, for example, as mitochondria are endowed with multiple signaling properties^4^. Alternatively, if some MSC mitochondria were damaged during the isolation process, acquisition of these dysfunctional mitochondria might trigger stress responses, like the mitochondrial unfolded protein response (mtUPR) and ultimately the MSC mitochondria elimination by mitophagy, leading to modification of the cellular processes^4^,^49^,^50^. Finally, we do not know the mechanisms that allow the transfer of the external MSC mitochondria inside the MDA-MB-231 cells. It is possible that during the process of cell internalization, the MSC mitochondria might trigger intracellular signaling pathways that are induced by the damage-associated molecular patterns (DAMPs), as mitochondria have recently been described as a source of such DAMPs that can activate diverse receptors, like the Toll-like receptors (TLRs) for example^51^,^52^,^53^. Obviously, answering these questions will require further investigation.

An intriguing observation was the low amount of mitochondrial DNA of MSC origin, detected by qPCR, in the MDA-MB-231 cancer cells after MitoCeption of the MSC mitochondria. As one might expect, and as we confirmed, the haplotypes of the mitochondrial DNA of the MSC donors and of the MDA-MB-231 patient were different and this could have an impact on the maintenance of the MSC mitochondrial DNA inside the MDA-MB-231 cells. Searching for MSC donors with the same haplotype as the MDA-MB-231 cancer cell donor could be difficult and therefore preclude us for testing this hypothesis in the MDA-MB-231 cell system. However, in a more general manner, our MitoCeption technique could be a powerful tool to study the processes involved in the regulation of heteroplasmy, a question central in the occurrence of metabolic diseases^54^,^55^.

In addition to our own data, other reports showed that isolated mitochondria can also be directly internalized by cells, not only *in vitro* but also *in vivo*^56^,^57^. These novel findings have several important implications. They highlight the existence of unexpected means of cell communication and signaling *in vivo* whose mechanisms and effects still need to be fully determined. In a methodological standpoint, the MitoCeption approach that we propose here provides valuable means to study the effects that the transferred mitochondria will have in the recipient cells, independently of the cytokine or metabolite signaling contribution.

When we tested the effects of the MSC mitochondria on the MDA-MB-231 cells, we observed a dose-dependent response with the maximal effects on ATP production and cell invasion for the intermediate amounts of 1.25 and 2.5 μg of MSC mitochondria. Likewise, the enhancement of the MDA-MB-231 proliferation capacities was maximal for the condition of 1.25 μg of MSC mitochondria. For all three measures, transfer of higher amounts of MSC mitochondria (i.e. 5 μg) did not give rise to effects of statistical significance. This could possibly be related to the opposite effects of the acquisition of the MSC mitochondria on the MDA-MB-231 OXPHOS and glycolysis rates. Indeed, both the aerobic glycolysis and the OXPHOS activity of the cancer cells have been related to their proliferation and metastasis capacity^8^,^58^. Therefore, one hypothesis would be that the decreased rate of glycolysis observed following the acquisition of higher amounts of MSC mitochondria might counter-balance the OXPHOS activity and be instrumental in the effects observed.

Concerning the interactions taking place in the tumor environment between MSCs and cancer cells, the occurrence of a transfer of mitochondria between these cell types constitutes an additional means, alternative to previously described processes, to modify the cancer cell metabolic and functional properties^6^,^58^,^59^. Diverse cell types are recruited to the tumor, among which endothelial cells have also been shown to transfer mitochondria to cancer cells^11^. It is not known so far whether a given cancer cell can be targeted for mitochondria transfer by both MSCs and endothelial cells and what would be the biological outcome of such combinatorial interactions. It is not known either whether repetitive mitochondria transfers to target cells might have cumulative effects. The MitoCeption technique should enable to address such questions. It should allow determining in particular whether mitochondria originating from diverse cell types generate different cellular outcomes. It should thus help establishing the effects that different cell types might have on cancer cell properties in the tumor microenvironment.

Because the mitochondrial activity is central to cell metabolism, a dysfunction in this activity can lead to severe diseases such as the Leber hereditary optic neuropathy (LHON), the Kearns-Sayre syndrome (KSS) or the mitochondrial recessive ataxia syndrome (MIRAS)^3^. The MitoCeption technique could enable the scientific community to study how disease-free mitochondria behave when introduced within a deficient mitochondria population. This could open new research areas and possibly lead to future therapeutic approaches. Cell metabolism is also central for cell fate decision and for embryonic and adult stem cell functions^60^. Thus, the MitoCeption technique could also be used to favor specific differentiation programs *ex-vivo* and have therapeutic applications, notably in the field of tissue engineering. Finally, because the mitochondria activity is affected during ageing, mitochondria failure could presumably play a key role in age-related diseases and tissue degenerescence^61^. MitoCeption could thus be used to supplement aged cells with young mitochondria and consequently allow the rejuvenation of the mitochondrial pool in cells ex-vivo. Such an approach could be used to rejuvenate cells for autologous cell-based therapies in regenerative medicine. Thus the applications of the MitoCeption technique are far-reaching, ranging from the understanding of fundamental biological mechanisms to devising novel therapies for metabolically based diseases.

## Methods

### Cell culture

Human MSCs were isolated from bone marrow aspirates from three healthy donors, each of whom gave informed consent. All the isolation and culture procedures were conducted in the authorized cell therapy unit (Biotherapy Team of General Clinic Research Center, French health minister agreement TCG/04/0/008/AA) at the Grenoble University Hospital. The cells were cultured in Minimum Essential Eagle Medium alpha (MEMα) supplemented with glutamine and 10% foetal calf serum (FCS) (all reagents from Invitrogen) and used at an early passage. All the hMSCs used were phenotyped by FACS analysis and their functionality was tested (colony-forming unit and *in vitro* differentiation in adipocyte and osteoblast) (data not shown). Cancer cells (MDA-MB-231 and TSA-pc) were grown in DMEM supplemented with FCS 10%. The cocultures were performed in DMEM/FCS 5% with MSCs seeded 24 hours before the addition of the cancer cells with equal numbers (10^5^) of each cell type in 35 mm dishes. When indicated, MSCs were MitoTracker labeled the day before (two days before the coculture), either with the green MitoTracker FM or the red MitoTracker CMXRos (Molecular Probes). Likewise, when indicated, the MDA-MB-231 cells were labeled with the MitoTracker CMXRos or with the green CellTracker CMFDA (Molecular Probes). All cells were trypsinized without EDTA in order to prevent membrane damage and MitoTracker leakage. Cell lines were checked to be negative for mycoplasma.

### Mitochondria isolation and transfer (Mitoception) to recipient cells

Cell and mitochondria exposure to EDTA was avoided at all steps of the protocol, therefore cells were trypsinized without EDTA and cocktails of protease inhibitors were also devoid of EDTA. In order to follow and quantify the rate and efficacy of the mitochondria transfer, mitochondria could be MitoTracker labeled beforehand in the donor cells. Likewise, the recipient cells could also be labeled (CellTracker). However, for the biological characterizations, MitoCeption was performed with unlabeled cells. Mitochondria were prepared using the Mitochondria Isolation Kit for Cultured Cells (Thermo Scientific) following the manufacturer's instructions. To obtain mitochondria preparations with reduced contamination from other cytosol compounds, centrifugation for the recovery of mitochondria was performed at 3,000 *g* for 15 minutes. Mitochondria preparations were done with 4 to 10.10^6^ MSCs. Typical yield in mitochondria was of 100 μg per million MSCs. The mitochondria preparation was resuspended in 1 ml of DMEM/FCS 5%, maintained on ice and used immediately for the artificial transfer.

The transfer of MSC mitochondria was performed on MDA-MB-231 cells seeded the day before (100 000 MDA-MB-231 cells per 22 mm-well). Before the transfer of the mitochondria isolated from the MSCs, MDA-MB-231 cells were counted from a control well and the amount of added mitochondria was adjusted accordingly. Throughout the manuscript, the amounts of mitochondria used refer to the amounts of MSC mitochondria (μg of proteins) for 100 000 MDA-MB-231 cells. The medium of the mitochondria recipient cells was changed to DMEM/FCS 5% and the mitochondria suspension was added slowly, close to the bottom of the well, throughout the culture surface. The culture plates were then centrifuged at 1,500 g for 15 min at 4°C. They were then placed in a 37°C cell incubator prior to a second centrifugation in the same conditions, two hours later. The following day, cells were rinsed with PBS and either analyzed by imaging or trypsinized for further testing.

### FACS analysis

Fluorescence Activated Cell Sorting (FACS) experiments were performed using a Becton Dickinson FACS-Canto II flow cytometer with 488-nm laser excitation and analyzed with CellQuest Pro software. Data are expressed with the mean fluorescence intensity (MFI) values.

### PCR analysis

Real time quantitative PCR was done on a Light Cycler 480 instrument (Roche, Meylan, France) using the SYBR green Master Mix (Roche, Meylan, France) and following the manufacturer's instructions. Nuclear DNA was quantified using the following primers nu-1: 5'- aca caa ctg tgt tca cta gc -3'; nu-2: 5'- cca act tca tcc acg ttc a -3', targeting the nuclear β-galactosidase gene. Mitochondrial DNA (mtDNA) was quantified by amplification of a DNA domain within the D-loop mt-1: 5'- tta act cca cca tta gca cc -3'; mt-2: 5'- gag gat ggt ggt caa ggg a- 3'^62^. To specifically quantify mtDNA from the MDA-MB-231 cancer cell, the reverse primer mt-2MDA: 5'- tta agg gtg ggt agg ttt gta ga -3' was used instead of mt-2. To specifically quantify mtDNA from MSCs, the reverse primer mt-2MSC: 5'- tta agg gtg ggt agg ttt gta gc -3' was used instead of mt-2. The amplification efficacies with the different sets of primers were verified with serial dilutions of the DNA from the MSCs and the MDA-MB-231 cells. In addition, to check the quantitative detection of MSC mtDNA in settings mimicking the MSC mitochondria transfer to the MDA-MB-231 cells, qPCR amplification of MSC mitochondrial DNA (with the MSC specific primers) was performed following serial dilutions of MSC DNA in a solution of MDA-MB-231 DNA.

### Extracellular Flux Analysis

The XF24 Flux analyzer (SeaHorse Bioscience) was used to measure oxygen consumption rate (OCR) and extracellular acidification rate (ECAR) on 100 000 MDA-231 cells, reflecting the rate of mitochondrial respiration and glycolysis respectively. All measures were performed 48 hours after the transfer of MSC mitochondria to the MDA-MB-231 cells.

OCR measurement was performed in XF media (nonbuffered DMEM) supplemented with 2.5 mM glucose, 2 mM L-glutamine and 1 mM sodium pyruvate, under basal conditions and in response to mitochondrial inhibitors: 1 μM oligomycin, 0.33 μM FCCP or 100 nM rotenone + 1 μM antimycin A (Sigma). Oxygen consumption rates (OCR, pMoles/min) were measured during 4 min. The basal respiration rate is calculated as the difference between basal OCR and OCR after inhibition of mitochondrial complex 1 and 3 with rotenone and antimycin A, respectively. The maximum respiration rate was measured following addition of the uncoupler FCCP (uncoupled rate), indicative of the maximum activity of electron transport and substrate oxidation achievable by the cells.

ECAR measurement was performed in XF media supplemented with 2 mM L-glutamine, in response to 10 mM glucose, 1 μM oligomycin and 200 mM 2-deoxyglucose (2-DG). The glycolytic capacity of the cells was calculated as the difference between the values of ECAR upon glucose addition and ECAR after 2-DG dependant inhibition of the glycolytic enzyme hexokinase. The glycolytic reserve was calculated as the difference between the value of ECAR upon glucose addition and ECAR following oligomycine-dependant inhibition of mitochondrial ATP synthase. It is indicative of the metabolic phenotype of the cells and their ability to shift from mitochondrial respiration to glycolysis in response to ATP demand. All SeaHorse measures were normalized to the number of cells counted in each well at the end of the SeaHorse experiments.

### ATP measurement

Measurements of the ATP produced by the control or MitoCepted MDA-MB-231 cells were performed on 50,000 cells, 48 hours after the transfer of MSC mitochondria, using the ATPlite luminescent detection assay, according to the manufacturer's instructions (Perkin Elmer). Measurements were expressed as Relative Luciferase Units (RLU) and calculated as fold of RLU measured in control MDA-MB-231 cells.

### Proliferation and invasion assays

For the proliferation assays, MDA-MB-231 cells with different amounts of MitoCepted MSC mitochondria were seeded in DMEM/FCS 5%, in quadruplicates, at the density of 10,000 cells per P24 well (5,000 cells/cm^2^) the day following MitoCeption. They were counted manually 5 days later.

Invasion assays of MDA-MB-231 cells were performed in 96-well View plates (PerkinElmer) pre-coated with 0.2% BSA (Sigma-Aldrich) and containing red fluorescent polystyrene microspheres at the bottom of the wells (10^4^ beads per well; FluoSpheres; Invitrogen). In brief, 24 hours after MitoCeption, the MDA-MB-231 cells (with or without added MSC mitochondria) were suspended in 1.7 mg/ml serum-free liquid bovine collagen at a concentration of 10^5^ cells/ml. 100-μl aliquots were dispensed into the plates. Plates were centrifuged at 300 g and incubated in a 37°C/5% CO_2_ tissue-culture incubator. Once collagen had polymerized, FCS was added on top of the collagen to a final concentration of 5%. After 72-h incubation at 37°C, cells were fixed in 4% formaldehyde (Sigma-Aldrich) containing Hoechst 33342 (5 μg/ml, Invitrogen). Confocal z slices were collected from each well at 0, 25 and 50 μm from the bottom (0 μm) using a high content microscope (Arrayscan VTI Live; Zeiss) with a 40× PlanFluor objective (0.5 NA; Zeiss). Nuclear staining in each slice was quantified automatically (Thermo Scientific Cellomics BioApplications-Image) to determine the percentage of invaded cells.

### Imaging

Fluorescence and time-lapse analysis was done with a Carl Zeiss LSM 5 live duo (LSM 510 META and 5 live) confocal laser system using a Zeiss 40× plan NeoFluar Oil objective. Time-lapse analysis was performed in an incubation chamber providing controlled temperature, CO_2_ concentration and hygrometry. Pictures were taken every 30 minutes for 24 to 36 hours. After imaging, all time points were compiled and exported as a Quicktime (avi) file using the MetaMorph software. For phase-contrast coupled to fluorescence microscopy, images were taken on a Carl Zeiss AxioVert 200 M inverted-phase microscope coupled to a Micromax YHS 1300 camera. Confocal imaging of the MDA-MB-231 cells containing the MitoTracker labeled MSC mitochondria, transferred by MitoCeption, was performed with a multiphoton Zeiss microscope LSM7. Imaging was done on the live cells in DMEM (without phenol red)/FCS 5%, with a 40× immersion objective. The 3D reconstructions were done using the Imaris Bitplane program.

For 3D-Structured Illumination Microscopy (3D-SIM) super-resolution imaging, the mitochondria preparation or the MSC cells were set on high precision #1.5H coverslips (Marienfeld) and mounted, respectively, in DMEM without phenol red or in Prolong Gold (Life Technologies). 3D-SIM imaging was performed on a OMX V3 microscope (GE) (Montpellier RIO Imaging), using 488 nm and 561 nm lasers, the corresponding dichroic and filter sets and an Olympus 100x/1.4 PSF grade oil immersion objective. Reconstruction and image registration of the 3D-SIM images were performed using softWoRx v5.9 (GE). 100 nm green and red fluorescent PS-speck beads (Life Technologies) were used to measure the optical transfer functions (otf), used for the 488 and 561 channels. 200 nm TetraSpeck beads (Life Technologies) were used to calibrate and verify the image registration quality.

For the transmission electron microscopy (TEM) analysis, 5 ml of the mitochondria preparation was dropped on a 150-mesh formvar-carbon copper grid for one minute and then negatively stained on a drop of uranyl acetate (1% in water). The copper grid was dried and analyzed at 75 KV using a Hitachi 7100 transmission electron microscope in the Resource Center for Cell Imaging (CRIC) of Montpellier, France. All digitalized images were mounted with the Adobe Photoshop software.

### Statistical analyses

Data were analyzed using GraphPad Prism 6 (GraphPad Software Inc., San Diego, CA). Multiple samples were analyzed by one-way analysis of variance (ANOVA) and Dunnett post hoc test to evaluate statistical differences among the samples. Differences were considered statistically significant for p < 0.05 (*P < 0.05, **P < 0.01, ***P < 0.001). All data are presented as mean values with S.E.M.

## Author Contributions

A.C. conceived the idea of MitoCeption. A.C., V.F., M.A., I.D. and N.A. performed the experiments. J.-M.B., C.A.P. and H.B. provided advice on technical developments. F.d.F. and A.M. supplied the human primary mesenchymal stem cells. A.C., V.F., J-M.B., C.J. and M.-L.V. designed the experiments and analyzed the data. M.-L.V. supervised the project and wrote the paper.

## Supplementary Material

Supplementary Information Supplementary data
