# Supplementary material for: MitoCeption as a new tool to assess the effects of mesenchymal stem/stromal cell mitochondria on cancer cell metabolism and function
Source: Sci Rep. 2015 Mar 13;5:9073. doi: 10.1038/srep09073 (PMC4358056; doi:10.1038/srep09073)

SUPPLEMENTARY DATA

MitoCeption as a new tool to assess the effects of mesenchymal stem/stromal cell mitochondria on cancer cell metabolism and functions

Andrés Caicedo[a,b], Vanessa Fritz[c], Jean-Marc Brondello[a,b], Mickaël Ayala[a,b], Indira Dennemont[a,b], Naoill Abdellaoui[a,b], Florence de Fraipont[d,e,f], Anaïck Moisan[d,e,g], Claire Angebault Prouteau[a,h], Hassan Boukhaddaoui[a,h], Christian Jorgensen[a,b,i] and Marie-Luce Vignais[a, b, j]

[a]CHU Saint Eloi, INM, 80 rue Augustin Fliche, 34295 Montpellier cedex 5, University of Montpellier 1, France, [b]Inserm U844, [c]Institut de Génétique Moléculaire de Montpellier, CNRS UMR 5535/IFR122, 1919 route de Mende, 34293 Montpellier Cedex 5, Universities of Montpellier 1 and Montpellier 2, France, [d]Univ Grenoble Alpes, 38000 Grenoble, France, [e]Inserm, U823 and U836, 38000 Grenoble, France, [f]Grenoble University hospital, Institut of Biologie and Pathology, 38000 Grenoble, France, [g]French blood company/Grenoble University hospital, Cell Therapy Unit, 38000 Grenoble, France, [h]Inserm U1051, [I]Department for Biotherapy at CHU Lapeyronie University Hospital, Montpellier, France.

[j]**Corresponding author :** Marie-Luce Vignais
Inserm U844, CHU Saint Eloi, INM
80 rue Augustin Fliche
34295 Montpellier cedex 5
France
E-mail: marie-luce.vignais@inserm.fr
Tel : (33) 4 99 63 60 27 / Fax : (33) 4 99 63 60 20

Supplementary data

Supplementary figure 1. Selected images from 3D-collagen cocultures of CellTracker stained MSCs (red) and MDA-MB-231 cancer cells (green) analyzed by real-time confocal imaging. MSCs and MDA-MB-231 were labeled beforehand, respectively with the red CMTPX and green CMFDA CellTrackers. The cocultures between the two cell types were done in a 3D-collagen 1 matrix and followed by time-lapse (Zeiss LSM510-live microscope) with images taken every 30 min for 24 to 36 h. Time T0 corresponds to the beginning of the time-lapse, 24 h after the beginning of the coculture **(a)** Cells displayed highly dynamic movements and were found to make physical contacts that could last for several hours as shown here for MSC (arrow) and MDA-MB-231 cells (arrow head). **(b)** During the time-lapse imaging, starting 24 hours after the beginning of the coculture, transfer of MSC cell components (marked by CellTracker vital dye) was observed at the early time-points (T0 to T8). Interestingly, the MDA-MB-231 cell with MSC cell components (indicated by the arrow) demonstrated a high migration capacity within the 3D collagen matrix in the 24 hours following the transfer (migrating cell indicated by the arrow heads). Similar exchanges of biological components were observed with the reverse CellTracker combination.

Supplementary figure 2. Transfer of hMSC mitochondria to murine cancer cells in coculture. After the coculture of murine TSA-pc cancer cells with human MSCs (prestained with red MitoTracker), human mitochondria were stained with the Ab-2 antibody (Ab-2 does not recognize murine mitochondria). In the overlay, murine cells are identified by their specific Hoechst staining pattern (scale bar, 20 mm). MSCs were observed to make long cell protrusions containing mitochondria (lower panel).

Supplementary figure 3. Coculture of MDA-MB-231 cells with MSCs MitoCepted with MSC mitochondria. To compare the properties of transferred versus endogenous mitochondria, we transferred mitochondria isolated from MSCs to other MSCs and asked whether these novel mitochondria also demonstrated the capacity to transfer to MDA-MB-231 cells in coculture conditions. For this purpose, red MitoTracker stained MSC mitochondria were transferred, through the MitoCeption protocol, to MSCs prestained with a green MitoTracker. Red MitoTracker-labeled MSC mitochondria were observed spread throughout the mitochondria network of these MSCs, thus validating the MitoCeption protocol. After the coculture (24h) between the MitoCepted MSCs and the MDA-MB-231 cancer cells, both the endogenous MSC mitochondria (green) and the exogenous MSC mitochondria (red) demonstrated the capacity to transfer to neighboring MDA-MB-231 cells. A confocal section (top panel) and 3D reconstructions of confocal image stacks (lower panels) are shown. Scale bars, 10 µm.

Supplementary figure 4. FCCP titration to obtain the maximal OCR capacity of the MDA-MB-321 cells. To determine the optimal concentration of the FCCP uncoupler in SeaHorse experiments, in order to obtain the maximal respiration capacity for each cellular condition (control and Mitocepted MDA-MB-231 cells), the OCR was measured with the XF24 Flux Analyser in response to increasing FCCP concentrations: 0.1 µM, 0.33 µM and 1 µM. All measures were normalized to the number of cells counted in each well at the end of the SeaHorse experiments. Measurements were performed in quadruplicates in 2 experiments. (a) Representative OCR graphs. (b) All values are shown as mean ± S.E.M.

Supplementary Figure 1

a

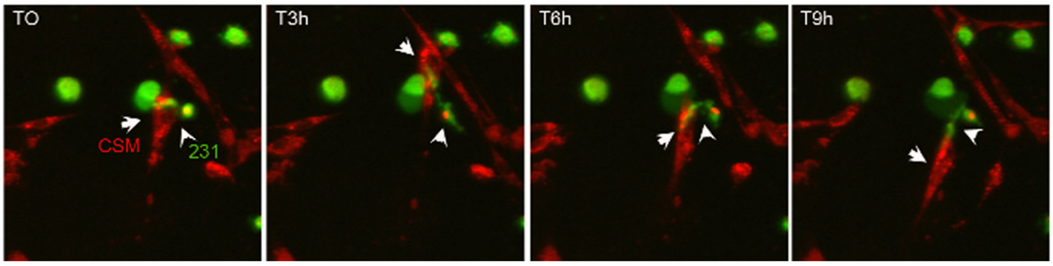

b

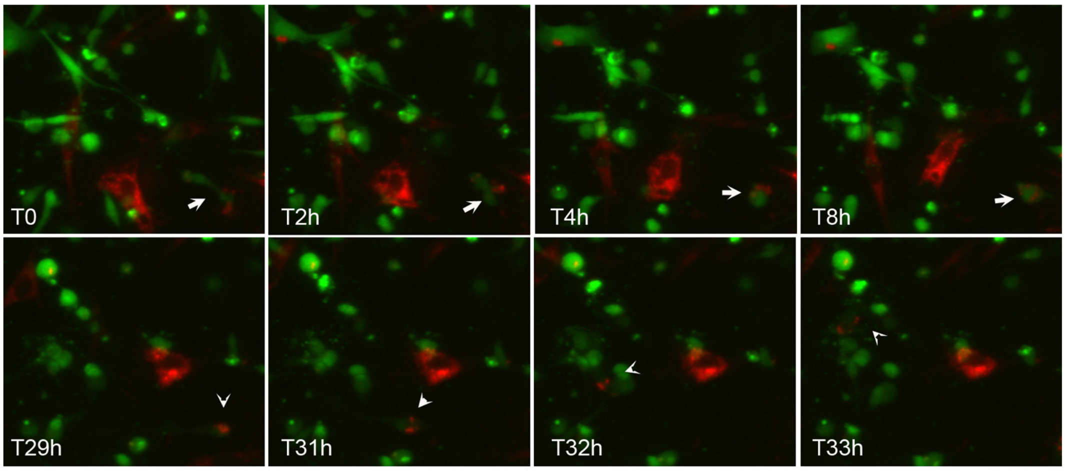

Supplementary Figure 2

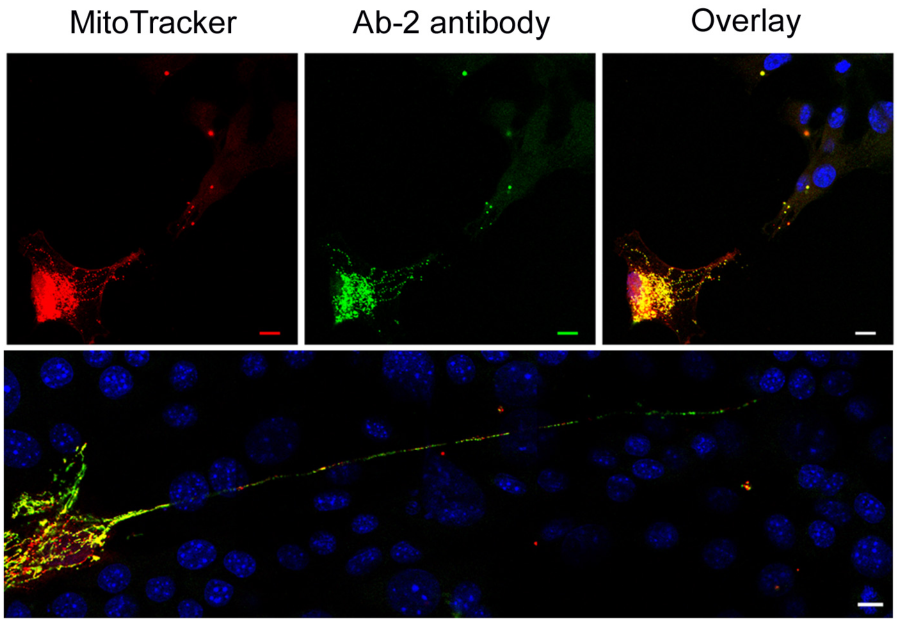

Supplementary Figure 3

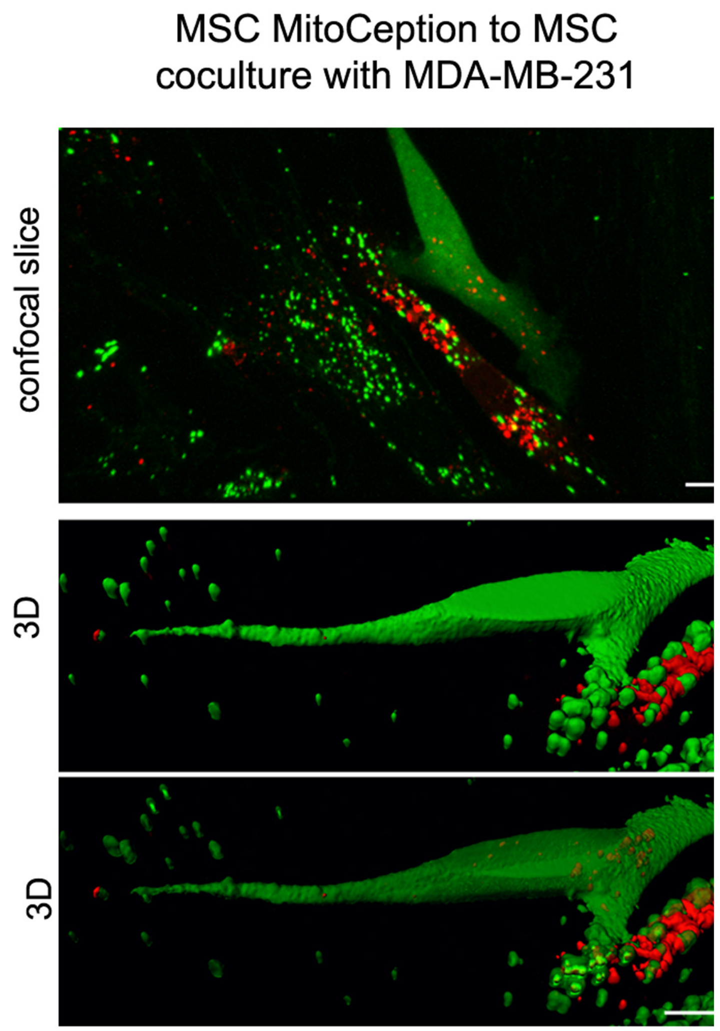

Supplementary Figure 4

a

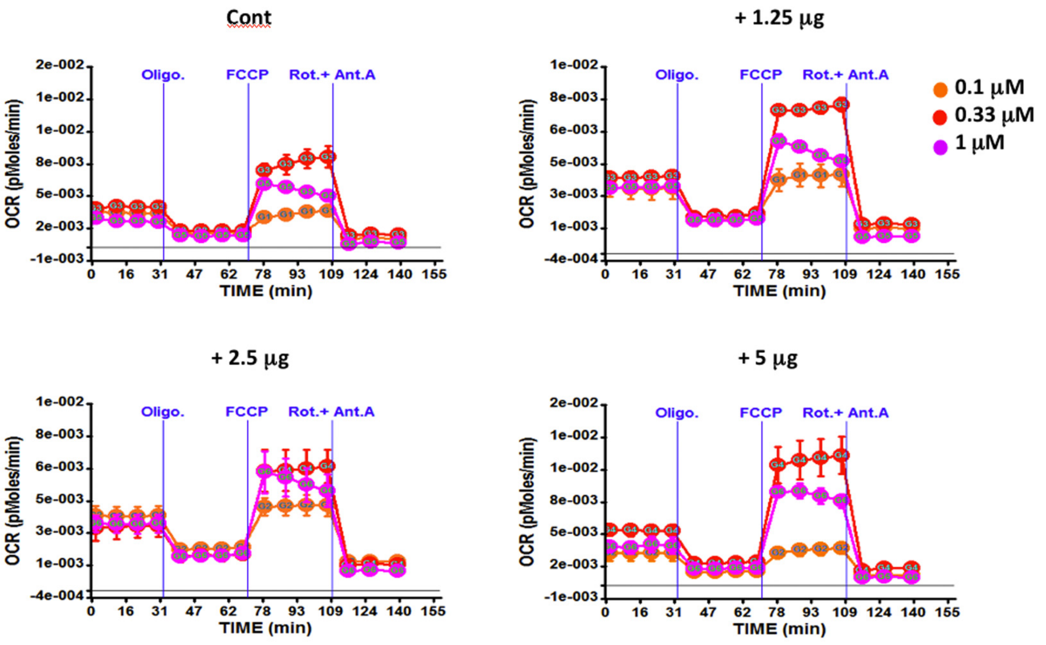

b

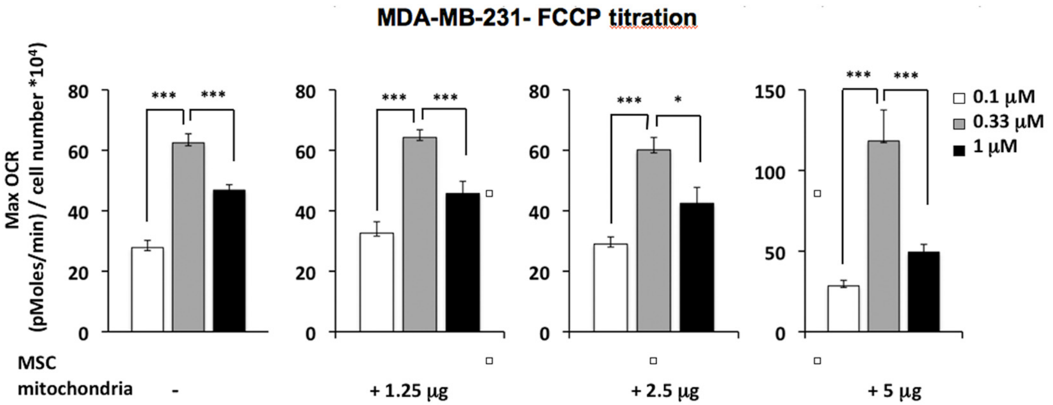

Supplement: Supplementary Information — Supplementary data [file srep09073-s1.pdf]
